# Predictors of Favourable Response to Cardiac Resynchronization Therapy in Patients with Heart Failure with Reduced Ejection Fraction: A Narrative Review

**DOI:** 10.3390/biomedicines14102239

**Published:** 2026-10-03

**Authors:** Magdalena Potapowicz-Krysztofiak, Małgorzata Maciorowska, Beata Uziębło-Życzkowska

**Affiliations:** Department of Cardiology and Internal Diseases, Military Institute of Medicine—National Research Institute, 04-141 Warsaw, Polandbuzieblo-zyczkowska@wim.mil.pl (B.U.-Ż.)

**Keywords:** cardiac resynchronization therapy, heart failure, mechanical dyssynchrony, predictors, responder, left bundle branch block

## Abstract

Cardiac resynchronization therapy (CRT) is an effective treatment for selected patients with heart failure with reduced ejection fraction, but 30–40% of recipients do not respond. This narrative review summarizes predictors of a favourable response to CRT. Typical left bundle branch block (LBBB) and a QRS duration ≥ 150 ms remain the best-established baseline predictors. Sex, etiology, age and comorbidities affect how much benefit is gained rather than acting as yes/no predictors (the benefit is greater in women, partly because of their smaller heart size) and should not by themselves exclude an otherwise eligible patient. QRS duration indexed to left ventricular size, advanced electrocardiographic markers (QRS notching, intrinsicoid deflection, QRmax, QRS area) and echocardiographic markers of mechanical dyssynchrony (apical rocking, septal flash, the classical LBBB strain pattern, systolic stretch index, mechanical dispersion and myocardial work) have been linked to response in observational studies and may add prognostic information. Combining mechanical dyssynchrony with septal viability on cardiac magnetic resonance appears to improve selection further. Procedure-related factors, including left ventricular lead position and a high percentage of biventricular pacing, are critical for success. A multimodal approach that combines clinical, electrocardiographic, echocardiographic and imaging data, supported in the future by machine learning, appears most promising.

## 1. Introduction

Cardiac resynchronization therapy (CRT) is an established treatment for symptomatic patients (New York Heart Association [NYHA] class II–IV) with heart failure with reduced ejection fraction (HFrEF) and dyssynchrony, despite optimal medical therapy. It was introduced in the 1990s [1], and its efficacy has been shown in many randomized trials. According to the 2021 ESC Guidelines on cardiac pacing and cardiac resynchronization therapy, CRT is indicated in patients in sinus rhythm who remain symptomatic (NYHA class II–IV) despite at least three months of optimized guideline-directed medical therapy and have a left ventricular ejection fraction (LVEF) ≤ 35%. The strongest (class I) recommendation applies to typical left bundle branch block (LBBB) on the electrocardiogram (ECG) with a QRS duration ≥ 150 ms; recommendations are weaker for other combinations of morphology and QRS duration [2]. Separate recommendations, not the main focus of this review, cover permanent atrial fibrillation (AF), patients with an indication for ventricular pacing and a high expected right ventricular pacing burden, and pacing-induced cardiomyopathy. The current recommendations are summarized in Table 1. Despite careful selection, better implantation techniques and optimal programming, about 30–40% of patients still do not respond (non-responders) [3]. The main causes include a left ventricular (LV) lead in a suboptimal position, particularly over transmural scar, absence of significant dyssynchrony, inappropriate atrioventricular and interventricular delays, and very advanced disease [4]. The devices are costly and carry risks such as lead infection, lead dislodgement, high pacing thresholds and phrenic nerve stimulation [5]. Good patient selection is therefore essential.

Assessment of dyssynchrony based on the ECG alone, and therefore only on electrical dyssynchrony, is insufficient, especially in ischaemic cardiomyopathy with an intermediate QRS duration of 130–150 ms. Much current research aims to identify more accurately the patients who will benefit from CRT. This review summarizes current knowledge on predictors of a favourable response to CRT. It was designed as a narrative, not a systematic, review. No protocol was registered, and no formal study-selection process or risk-of-bias assessment was applied.

Relevant articles were identified by searching PubMed and Google Scholar from database inception up to September 2026. The full search strategy, including the search strings and the inclusion and exclusion criteria, is given in Supplementary Materials S1. Overlapping cohorts are identified in the text and tables.

Articles were selected on the basis of clinical relevance and the authors’ expertise, with priority given to randomized trials, patient-level meta-analyses and multicentre studies. This approach gives a broad overview of a fast-moving field but carries a risk of selection bias. The framework of the review is summarized in the Central Illustration. The evidence points to one organizing principle: CRT works best when mechanical dyssynchrony is caused by delayed electrical activation and the myocardium involved is viable; it fails when dyssynchrony is absent, is not electrically driven, or when the septum is scarred. The parameters discussed below are treated as refinements of this principle rather than as independent predictors.

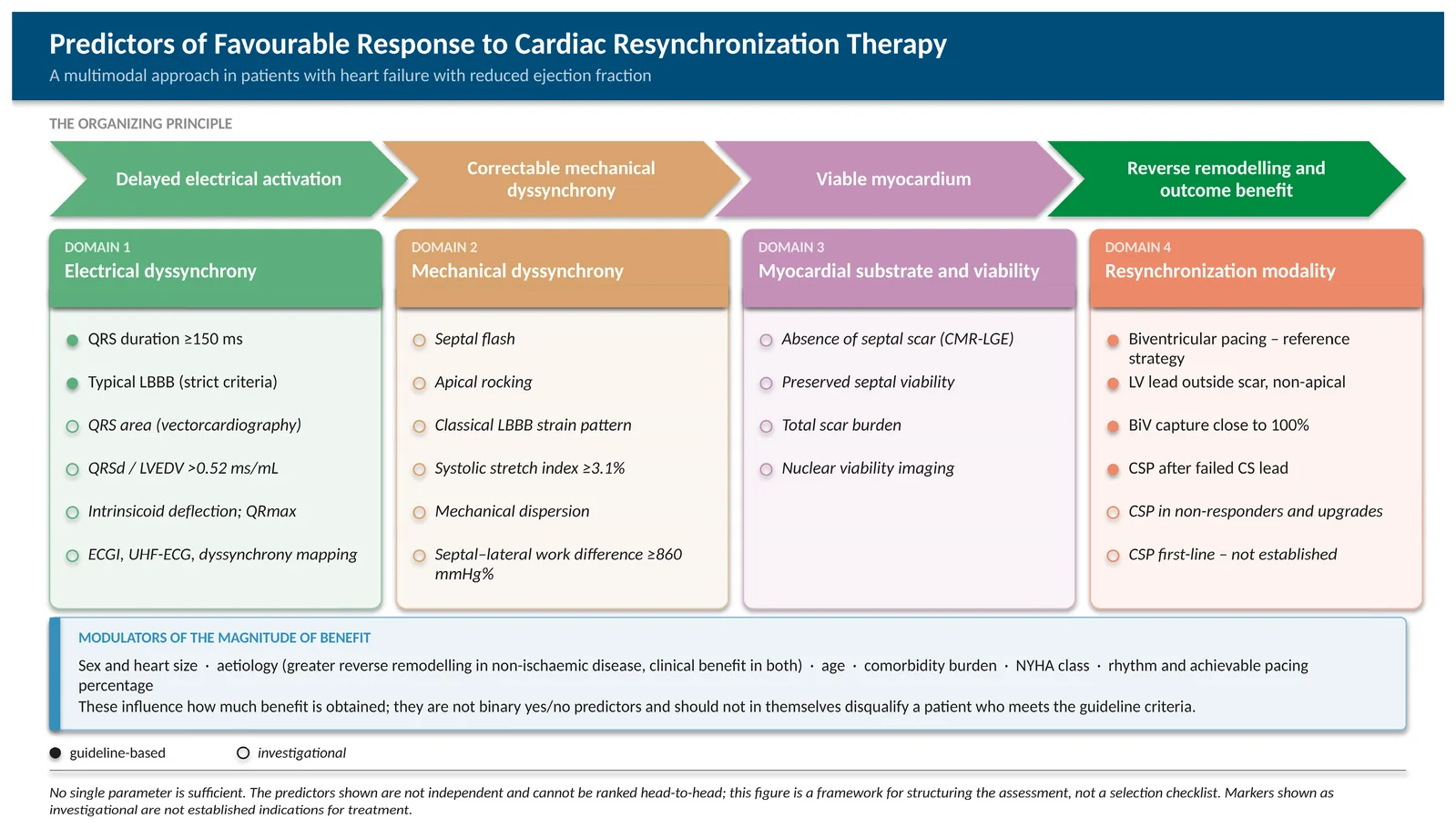



**Central Illustration.** Predictors of a favourable response to cardiac resynchronization therapy. The top band shows the organizing principle of the review: CRT works best when delayed electrical activation causes a correctable mechanical dyssynchrony in viable myocardium. The four panels show the domains assessed: electrical dyssynchrony, mechanical dyssynchrony, myocardial substrate and viability, and choice of resynchronization modality. Filled markers show parameters that are part of current guideline recommendations; open markers in italics show investigational parameters that are not established indications for treatment. The lower band lists factors that change the size of the benefit rather than acting as yes/no predictors. Abbreviations: BiV, biventricular; CMR, cardiac magnetic resonance; CRT, cardiac resynchronization therapy; CS, coronary sinus; CSP, conduction system pacing; ECGI, electrocardiographic imaging; LBBB, left bundle branch block; LGE, late gadolinium enhancement; LV, left ventricle; LVEDV, left ventricular end-diastolic volume; NYHA, New York Heart Association; QRSd, QRS duration; UHF-ECG, ultra-high-frequency electrocardiography.

## 2. The Role of Dyssynchrony

The degree of LV dyssynchrony is considered one of the most important determinants of CRT efficacy. Dyssynchrony plays a key role in heart failure (HF), affecting LV systolic and diastolic function as well as right ventricular (RV) and left atrial (LA) function. It has an electrical component, related to delayed intraventricular conduction and a prolonged QRS, and a mechanical component, reflecting non-simultaneous activation, shortening and stretching of LV segments [6].

Clinically, dyssynchrony can be atrioventricular, interventricular or intraventricular. Atrioventricular dyssynchrony mainly impairs diastolic filling, because ventricular contraction starts before diastole is complete; this shortens filling time and may worsen mitral regurgitation. Many patients with HFrEF have intraventricular conduction disease, especially LBBB. This causes electrical and then mechanical dyssynchrony, which worsens systolic function, increases mitral regurgitation and promotes adverse LV remodelling. Because CRT is an electrical therapy, its primary aim is to correct electrical dyssynchrony; mechanical improvement follows from a more normal activation sequence [7].

In well-selected patients, CRT leads to reverse remodelling, better exercise tolerance and quality of life, fewer HF hospitalizations and lower mortality. Fewer ventricular arrhythmias have also been reported, attributed to reverse remodelling itself. This should be distinguished from the effect of the defibrillator. Adding an implantable cardioverter-defibrillator (CRT-D) serves a separate indication, the prevention of sudden arrhythmic death. CRT-D and CRT-P have not been compared in an adequately powered randomized trial, so the choice between them should follow the current ICD indications and is not a determinant of the resynchronization response. CRT is less effective when mechanical dyssynchrony is caused by inflammation, fibrosis, ischaemia or post-infarction scar.

## 3. Definition of Response to CRT

There are no universally accepted criteria for defining response to CRT. Large trials used different criteria, which limits comparison of their results. Response can be assessed by clinical, electrocardiographic, echocardiographic or biochemical criteria (e.g., N-terminal pro–B-type natriuretic peptide [NT-proBNP]), usually 6–12 months after implantation [8]. In practice, clinical and echocardiographic criteria are used most often. The most common echocardiographic criteria are a reduction in LV end-systolic volume (LVESV) of at least 10–15% or an increase in LV ejection fraction (LVEF) of at least 5–10%, reflecting reverse remodelling. Greater reverse remodelling is associated with better prognosis and fewer HF hospitalizations [9].

Clinical response is usually defined as improvement by at least one NYHA class, fewer HF hospitalizations, or a composite endpoint. QRS narrowing after implantation, often by at least 20 ms, has been studied as an electrical marker of response [10]. The final QRS width and shape depend not only on baseline QRS width but also on lead position and pacing settings. Because HF is a multifactorial, progressive disease, the main therapeutic target should be identified before starting therapy. Figure 1 summarizes the predictors of favourable and unfavourable response to CRT.

Because these endpoints are not interchangeable, the following terms are used throughout this review. “Echocardiographic response”, used as a synonym of “reverse remodelling”, means a reduction in LVESV of at least 10–15% and/or an increase in LVEF of at least 5–10% at 6–12 months. “Clinical response” means improvement by at least one NYHA class, or better exercise capacity or quality of life. “Outcome benefit” means fewer HF hospitalizations, lower cardiovascular or all-cause mortality, or a composite of these. “Electrical response” means QRS shortening. The term “responder” is used only when the cited study used a yes/no classification, and its criterion is stated. When studies with different endpoints are discussed together, the endpoint of each is given, because agreement between the common definitions is poor [8], and a parameter that predicts reverse remodelling does not necessarily predict survival. Table 2 summarizes the definitions of response used in the key studies.

Biochemical response, usually a fall in NT-proBNP, is mentioned because it appears among the response definitions in the literature, but it is not discussed further. It is reported much less consistently than the other endpoints; there is no agreed threshold, and its predictors have not been studied separately from those of echocardiographic or clinical response. The endpoint used for each parameter is given in the “Type” column of Table 3 and Table 4 and in the “Endpoint” column of Table 5, and sensitivity, specificity and cut-off values in the text are given with the population in which they were obtained. A parameter that predicts reverse remodelling should not be assumed to predict better survival.

## 4. Clinical and Demographic Factors

Randomized trials and meta-analyses suggest that CRT benefits women more than men [26]. Women have fewer HF hospitalizations and lower mortality, more often have LBBB and non-ischaemic cardiomyopathy (NICM), and less often have AF, all of which are associated with a better response. For the same QRS duration, they have more baseline dyssynchrony, a larger QRS area and more often the classical LBBB contraction pattern, and the benefit of CRT is also greater in shorter patients of either sex [27,28,29].

This suggests that heart size, rather than sex itself, may be the key variable, and several groups have tested whether indexing QRS duration to body or heart size improves selection. In a patient-level meta-analysis of seven trials (5628 patients, 25.6% women), CRT reduced all-cause mortality from a QRS duration of about 137 ms in women versus about 164 ms in men; indexing QRS duration to height, body surface area (BSA) or LV end-diastolic diameter did not fully remove this difference, and the median LVEF gain at one year was larger in women (11% vs. 8%; *p* < 0.001) [23]. Indexing to LV end-diastolic volume performed better: a QRSd/LVEDV above 0.52 ms/mL identified patients with more than twofold better survival and was an independent prognostic factor, and the sex differences were largely explained by heart size and indexed QRS duration [14,30]. The same index predicted CRT efficacy in patients with an intermediate QRS duration of 120–149 ms [25].

Indexing QRS duration to heart size therefore seems most useful where the standard criteria help least (borderline QRS duration and non-ischaemic etiology), but it explains only part of the sex difference.

Whether similar differences exist between racial or ethnic groups is much less clear, mainly because non-white patients have been under-represented in the pivotal trials [26]. In MADIT-CRT, only 143 of 1781 patients (8%) were Black [31]. They had more diabetes and treated hypertension and a worse prognosis, with a higher three-year risk of HF or death, but their echocardiographic response to CRT-D and the relative risk reduction did not differ significantly from those of white patients. Registry data [32] suggest that the main disparity is in access to therapy rather than in response, since eligible Black and Hispanic patients are less likely to receive a CRT device than white patients. Given the small numbers, no firm conclusion can be drawn about ethnic differences in response, and studies in more diverse populations are needed.

The etiology of HF also matters. In an analysis by Sudesh et al. of 6252 patients from seven randomized trials (MIRACLE, MIRACLE-ICD, MIRACLE-ICD II, COMPANION, REVERSE, MADIT-CRT and RAFT), CRT efficacy was largely independent of etiology. Patients with NICM showed greater improvement in LVEF and LV dimensions, suggesting more reverse remodelling, but both groups had fewer hospitalizations and lower mortality. The mechanism of benefit may therefore differ by etiology: in NICM, reverse remodelling appears to dominate, whereas in ischaemic cardiomyopathy (ICM), slowing of progressive adverse remodelling may be more important [24].

Age and comorbidity burden appear to be weaker predictors. A meta-analysis of eight trials (BLOCK-HF, MIRACLE, MIRACLE-ICD, MIRACLE-ICD II, RAFT, COMPANION, MADIT-CRT and REVERSE) showed that CRT improves outcomes regardless of comorbidity burden [33], although more comorbidities were associated with a worse prognosis. Older age and comorbidities should therefore not by themselves exclude patients from CRT [34]. In a meta-analysis by Martins et al., NYHA class II was an independent predictor of response [35].

## 5. Electrocardiographic Predictors

### 5.1. QRS Morphology and Duration

The 12-lead ECG is practical, inexpensive and widely available, and CRT indications are based mainly on QRS duration and morphology. QRS duration is an independent risk factor for all-cause mortality and sudden cardiac death (SCD), particularly in ischaemic etiology [36]. Many studies have shown that patients with LBBB respond more often than those with other morphologies (non-LBBB). However, about one third of patients with LBBB by standard criteria may not have truly delayed LV conduction, for example, because of hypertrophy, LV dilatation or left anterior fascicular block [37,38]. In a 2023 meta-analysis of 6264 patients from eight randomized trials, Friedman et al. found the greatest benefit in patients with LBBB and a QRS duration ≥ 150 ms, although benefit may start at about 130 ms. In non-specific intraventricular conduction delay (IVCD), benefit was seen only at a QRS duration ≥ 150 ms, and in right bundle branch block (RBBB) there was no significant clinical benefit, probably because significant LV dyssynchrony is absent. RBBB and IVCD should therefore not be treated as one “non-LBBB” group when selecting patients [19].

The definition of LBBB differs between societies. In an analysis of 1492 pre-implantation ECGs, only 13.8% of patients met all tested LBBB definitions (ESC, AHA/ACCF/HRS, MADIT-CRT, REVERSE and Strauss et al.) [39]. Whatever the criteria, features associated with a favourable response were a QS or rS complex in V1, notching or slurring in V5, V6, I or aVL, and no Q waves in these lateral leads.

Because LBBB morphology predicts benefit best when conduction delay is truly present, we recommend the strict criteria of Strauss et al. when morphology is used to predict response. However, the guideline indications are based on the looser definitions used in the pivotal trials. A patient can therefore have a class I indication without meeting the definition supported by the predictive evidence. This gap is often overlooked and should be kept in mind when morphology-based predictions are applied to an individual patient.

In a recent Dutch study, typical LBBB and a QRS duration > 150 ms were the only significant ECG predictors of response [40]. In contrast, a very long QRS (≥178 ms) in LBBB was associated with less echocardiographic response and a worse prognosis than a moderately wide QRS [41]. Additional ECG markers may therefore be most useful in patients without typical LBBB.

### 5.2. Other Electrocardiographic Markers

Patients with left axis deviation more often have a shorter LV activation time, more scar in the apical and anteroseptal segments, and more hypertrophy [42]. In LBBB, left axis deviation has also been associated with a worse prognosis, more fibrosis and hypertrophy, and a shorter activation time, while patients with an intermediate axis appear to benefit more from CRT [43].

In a large multicentre retrospective Italian study of 1718 patients, echocardiographic response at 6 months was more frequent with a PR interval up to 160 ms [44]. In multivariable analysis, a PR interval of 150–170 ms was associated with a higher likelihood of response and a lower rate of death or HF hospitalization. In contrast, Kutyifa et al. found that in patients without LBBB, a long PR interval (>230 ms) was associated with better outcomes than a normal PR interval [45].

QRS notching may add information beyond QRS duration and morphology. Bertaglia et al. [46] and Pan et al. [47] showed that notching in at least one lateral lead in LBBB was independently associated with echocardiographic response. Kodama et al. showed that patients with LBBB and an RR′ duration ≥ 48 ms had more mechanical dyssynchrony, more viable myocardium and better survival after CRT [48].

Other QRS features have been linked to response in single cohorts: the S-wave amplitude in the right precordial leads, the R-peak-to-S-nadir interval in V1 and its shortening after implantation, an S-wave and the R/S ratio in lead V6, and the lead one ratio. Their cut-offs, endpoints and cohorts are shown in Table 3 [49,50,51,52,53].

### 5.3. Intrinsicoid Deflection and Other Signs of LV Activation

Some ECG parameters may show delayed LV activation even without LBBB morphology. The time to the R-wave peak in lateral leads (≥110 ms in lead I, ≥130 ms in aVL, the difference between leads I and V1, and a lead I time/QRS ratio > 0.69) has shown prognostic value in LBBB or IVCD [54]. Vereckei and Katona developed two indices that express, as a percentage of QRS duration, the difference in intrinsicoid deflection time between the lateral and inferior walls and between the left and right ventricles (Table 3). Values > 25% for both indices were associated with a higher likelihood of response [55].

In patients who do not meet the Strauss criteria for “true LBBB” [37], a long Q–LV interval was associated with mid-QRS notching and/or slurring, a QRS duration > 150 ms and an ID > 60 ms in V6. Patients with atypical RBBB also had a long Q–LV interval (>110 ms), identifying a subgroup with delayed LV free-wall activation that may respond to CRT [56]. A later study by the same group confirmed that patients with RBBB and delayed LV activation can benefit from CRT [57].

Another indirect marker of intraventricular dyssynchrony is the QRmax index, the longest interval in the limb leads from QRS onset to the end of the R wave. A QRmax > 120 ms correlates better with the Q–LV interval than QRS duration does, and in its derivation cohort it predicted echocardiographic response better than QRS duration [58]; this has not been tested in another population. Vectorcardiographic QRS area reflects global electrical dyssynchrony, taking into account both the amplitude and the spatial direction of depolarization. Higher values are linked to a better response, and a meta-analysis of observational cohorts found that QRS area separated responders better than QRS duration and LBBB morphology [59]; this has not been tested prospectively. Newer methods include vectorcardiography (VCG), ultra-high-frequency ECG and electrical dyssynchrony mapping, and more advanced techniques such as electrocardiographic imaging (ECGI) and electroanatomical mapping; they are promising but not yet in routine use. Figure 2A,B show ECGs before and after CRT in a patient with a favourable response. The cut-off values in this section come mostly from single, small cohorts and were derived within those cohorts (Table 3); they are indicative and not thresholds for clinical decisions.

**Table 3:** **Electrocardiographic predictors of response to cardiac resynchronization therapy.** Type of response: E, echocardiographic (LVESV reduction ≥15%); O, outcome (death and/or HF hospitalization).

Parameter	Definition/Cut-Off	Reported Association	Cohort and Validation Status	Type	Ref.
**QRS duration**	• ≥150 ms vs. 130–149 ms	• Greatest benefit ≥ 150 ms • Benefit may start from ≈130 ms	Narrative review [36]; *n* = 243, retrospective, threshold derived within the cohort [41]. The ≥150 ms partition itself was pre-specified and tested in a patient-level meta-analysis of 8 RCTs (*n* = 6264) [19].	O	[19,36,41]
**QRS morphology**	• Typical LBBB (Strauss criteria)	• Best-established ECG predictor • No benefit in RBBB • In IVCD only at ≥150 ms	Consensus definition with simulation data [37]; morphology-stratified effect confirmed in a patient-level meta-analysis of 8 RCTs (*n* = 6264) [19]. Guideline criterion.	O	[19,36,37,41]
**QRS indexed to LV size**	• QRSd/LVEDV > 0.52 ms/mL	• Independent predictor of survival • Useful in QRS 120–149 ms	*n* = 3739, post hoc analysis of an RCT [30]; *n* = 250, two centres [14]; *n* = 119, multicentre retrospective, derivation only [25].	O	[14,25,30]
**QRS axis**	• Axis deviation with LBBB	• Left or right axis deviation: worse outcome • Left axis deviation: more scar and hypertrophy, less LV activation delay • Intermediate axis favourable	*n* = 707, multicentre retrospective [42]; *n* = 68, prospective [43]. Conventional axis categories; no threshold derived.	O	[42,43]
**PR interval**	• 150–170 ms • ≥230 ms in non-LBBB	• Higher likelihood of response • In non-LBBB, PR > 230 ms: better outcome	*n* = 1718, observational registry, derivation only [44]; *n* = 537 non-LBBB, post hoc MADIT-CRT analysis, derivation only [45].	E, O	[44,45]
**QRS notching**	• Mid-QRS notching or slurring in ≥1 lateral lead • RR′ ≥ 48 ms	• Independent predictor of response • RR′ ≥ 48 ms also longer survival	*n* = 335 with LBBB, prospective multicentre registry, applying pre-existing Strauss criteria [46]; *n* = 82, single centre [47]; cohort size not reported in [48], threshold derived within that cohort.	E, O	[46,47,48]
**S wave, right precordial leads**	• S amplitude > 1.44 mV in V1–V3 • R peak to S nadir in V1 > 45 ms • Shortening > 10 ms after CRT	• Favourable response	*n* = 47, single-centre retrospective, derivation only [49]; *n* = 51, single-centre prospective, derivation only [50].	E	[49,50]
**S wave and R:S ratio, lead V6**	• Absence of deep S wave in V6 • Higher R/S ratio in V6	• S wave in V6 predicts non-response • Higher R/S ratio: more favourable response	*n* = 112 with LBBB, multicentre retrospective [51]; *n* = 102, single-centre prospective [52]. Binary variables; no threshold derived.	E	[51,52]
**LOR**	• Maximum positive/negative QRS deflection in lead I >12	• Favourable clinical and echocardiographic response	*n* = 93, single-centre prospective; threshold applied within the cohort, derivation only [53].	E, O	[53]
**ID**	• ≥110 ms in lead I • ≥130 ms in aVL • ID/QRS in lead I >0.69 • >60 ms in V6	• Predicts LV reverse remodelling • Correlates with prolonged Q–LV	*n* = 135, single centre; all thresholds derived within the cohort, no validation set [54].	E	[54]
**Vereckei dyssynchrony indices**	• [aVL-ID—aVF-ID]/QRSd >25% • [V5-ID—V1-ID]/QRSd >25%	• Higher likelihood of favourable response	*n* = 124, single-centre retrospective. The >25% threshold was derived in a separate set of 68 patients and then tested in this cohort—the only independently tested threshold in this table [55].	E, O	[55]
**QRmax index**	• >120 ms	• Correlates with Q–LV better than QRS duration • Better predictor of response than QRS duration in the derivation cohort	*n* = 173 without LBBB, observational; threshold derived within the cohort [58].	E	[58]
**QRS area (VCG)**	• Higher values	• May outperform QRS duration and LBBB morphology	Systematic review and meta-analysis [59]; pooled cohort size not stated in the available abstract. Thresholds differ between the contributing cohorts.	E, O	[59]
**Atypical RBBB with delayed LV activation**	• RBBB without wide terminal negative deflection in lateral limb leads • Q–LV >110 ms	• Identifies RBBB patients who can benefit from CRT	*n* = 192, two-centre observational, derivation only [56]; *n* = 66, multicentre retrospective [57].	E	[56,57]

CRT, cardiac resynchronization therapy; ECG, electrocardiographic; HF, heart failure; ID, intrinsicoid deflection; IVCD, non-specific intraventricular conduction delay; LBBB, left bundle branch block; LOR, lead one ratio; LV, left ventricular; LVEDV, left ventricular end-diastolic volume; LVESV, left ventricular end-systolic volume; QRSd, QRS duration; Q–LV, interval from QRS onset to left ventricular electrogram; RBBB, right bundle branch block; VCG, vectorcardiography.

## 6. Echocardiographic Predictors

Despite progress in echocardiography, its role in CRT selection still relies mainly on LVEF. Many echocardiographic indices of mechanical dyssynchrony have been studied, but large randomized trials such as PROSPECT and EchoCRT questioned their clinical value [60,61], and in patients with a narrow QRS, CRT guided by these indices was even harmful [62]. However, these trials had methodological limitations and mainly used standard parameters rather than modern techniques. These parameters also depended strongly on loading conditions.

LV apical rocking (ApRock), septal flash (SF), early systolic septal motion, and systolic stretch of the septum and lateral wall are well-known features of mechanical dyssynchrony that help predict response and mortality after CRT [12]. The PREDICT-CRT study confirmed the prognostic value of visually assessed apical rocking and septal flash. These septal and apical motion abnormalities are part of the typical LBBB contraction pattern and share one mechanism. They should complement, not replace, the classical selection criteria [63]. A small single-centre study showed that semi-automated detection of these patterns by speckle-tracking echocardiography (STE) was more accurate than visual assessment by less experienced readers [64].

Risum et al. showed that a characteristic LV strain pattern may be an important predictor of CRT efficacy [11]. It is assessed from longitudinal strain curves of LV segments obtained by STE in three apical views. In LBBB, a typical sequence of wall activation can be seen, which the authors called the classical LBBB strain pattern. It requires three features in at least one apical view: early shortening of at least one basal or mid septal segment with stretching of at least one basal or mid lateral segment; early septal contraction within the first 70% of the ejection period; and peak lateral wall strain after aortic valve closure. The presence of this pattern suggests a conduction abnormality that CRT can correct. In that cohort, the pattern predicted echocardiographic response (LVESV reduction ≥ 15% at six months) with a sensitivity of 95% and a specificity of 91% [11]. These values refer to reverse remodelling, not to survival.

In the STE-based LBBB classification of Calle et al., types LBBB-3/4 were associated with marked LBBB-induced remodelling and predicted a favourable response, including super-response, after CRT [65]. Aalen et al. showed that lateral wall scar leads to loss of septal rebound stretch, whereas extensive anterior ischaemia increases it [66]. Like the myocardial work thresholds discussed below, the values reported by Risum et al. come from a single prospective cohort of 67 patients in which the pattern was both defined and tested, with no validation cohort. The classification of Calle et al. was also derived within its own cohort.

The systolic stretch index (SSI) is another strain-based parameter: the sum of posterolateral wall stretch before aortic valve opening and septal rebound stretch during ejection. An SSI ≥ 3.1% was associated with freedom from HF hospitalization or death within two years after CRT, and favourable outcomes were seen even with an SSI ≥ 2.6% in patients with a QRS of 120–149 ms [15].

LV mechanical dispersion is another marker of uneven timing of contraction and a possible predictor of response. It quantifies the severity of mechanical dyssynchrony and is calculated as the standard deviation of the time to peak negative longitudinal strain across LV segments. Greater baseline dispersion has been associated with a higher likelihood of response and improved LV function after CRT [67]. It may be most useful in patients without typical LBBB, in whom QRS morphology and width alone may not be enough [68].

Myocardial work (MW) is promising in CRT but remains investigational: the evidence comes almost only from observational, mostly single-centre cohorts, with cut-offs defined post hoc and no prospective external validation. These parameters are therefore associated with response rather than determinants of it, and none is part of guideline-based selection. The rationale is mechanical: when conduction is delayed, the early-activated septum contracts before aortic valve opening and stretches the lateral wall, and the late lateral contraction then stretches the septum, so part of the energy is spent on deforming opposite segments instead of on ejection.

Combining a non-invasive pressure curve with segmental strain (the pressure–strain loop, PSL) provides measures of this as global constructive work (GCW), which contributes to ejection, and global wasted work (GWW), which does not, together with global work efficiency (GWE) and global work index (GWI).

Unlike classical dyssynchrony indices, this work correlates with invasively measured work and with residual metabolic activity on FDG positron emission tomography in CRT candidates [69], and the constructive work left in the dyssynchronous ventricle is thought to reflect the contractile reserve available for recovery.

In a prospective multicentre study of 200 CRT patients, Aalen et al. [17] assessed myocardial work and viability by cardiac magnetic resonance (CMR). Work asymmetry with reduced septal work but preserved septal viability was associated with more reverse remodelling. In this cohort, a difference in work between the lateral wall and the septum of at least 860 mmHg% identified echocardiographic responders with an accuracy of 75% and was also associated with lower mortality; these are two different endpoints in the same population. Septal scar on CMR identified non-responders with a sensitivity of 81%, again for the echocardiographic endpoint. In multivariable analysis, the work difference, HF etiology, septal viability and a QRS duration of 120–150 ms (but not LBBB morphology) were independent predictors of reverse remodelling. In that cohort, the work difference separated responders better than QRS duration and morphology, septal flash or apical rocking. This is a comparison within one study and does not establish a general ranking of these markers.

Galli et al. reported that constructive and wasted work are related both to echocardiographic response and to the degree of remodelling after CRT, and that low constructive work was associated with cardiac death [13,70,71]. These three reports are not independent: Refs. [13,71] describe the same single-centre cohort of 97 patients (63 responders), and Ref. [70] describes an expanded cohort of 166 patients from the same centre. Their agreement therefore reflects one population, not replication across centres. Riolet et al. found that low global wasted work, absence of LBBB and significant scar were independently associated with non-response and higher all-cause mortality, whereas septal flash and apical rocking were not [18]. Van der Bijl et al. reported that lower work efficiency before implantation was independently associated with better long-term outcome in patients with a class I indication, with a trend towards higher risk at the lowest values, possibly reflecting exhausted contractile reserve [16]. The thresholds, cohorts and endpoints are given in Table 4.

In patients with a wide QRS but no typical LBBB, Layec et al. found a better response with a GWW above 200 mmHg% [20], consistent with the finding of Riolet et al. that a GWW below the same value marks non-response and higher mortality. In the study of Layec et al., the ROC-optimal value was 183 mmHg%, rounded to 200. Both thresholds were derived post hoc within their own cohorts, and no prospective study has shown that selecting patients by myocardial work improves outcomes. The acute shift in regional work after CRT, with more septal and less lateral work, has been proposed as a determinant of long-term reverse remodelling [72].

A meta-analysis also showed that baseline RV function, especially tricuspid annular plane systolic excursion (TAPSE), may predict response to CRT and should be considered in future selection algorithms [73].

Figure 3A,B show examples of a favourable and an unfavourable echocardiographic response to CRT.

**Table 4:** **Echocardiographic predictors of response to cardiac resynchronization therapy.** Type of response: E, echocardiographic (LVESV reduction ≥15%); O, outcome (death and/or HF hospitalization).

Parameter	Definition/Cut-Off	Reported Association	Cohort and Validation Status	Type	Ref.
**Eligibility**					
**LVEF**	• ≤35%	• Eligibility criterion rather than a predictor of response	Guideline eligibility criterion [2].	—	[2]
**Visual and deformation markers of dyssynchrony**					
**Septal flash and apical rocking**	• Visual assessment • Semi-automated detection by STE	• Predict response and long-term survival (PREDICT-CRT) • Complementary to classical criteria	*n* = 1060, multicentre observational (PREDICT-CRT); sensitivity and specificity reported within that cohort [12]. Narrative review [63]. *n* = 125, two centres, semi-automated detection derived in 25 patients and applied to the remaining 100 (split-sample internal validation) [64].	E, O	[12,63,64]
**Classical LBBB strain pattern**	• Early septal shortening with lateral stretch • Early septal contraction within first 70% of ejection • Peak lateral strain after AVC • All three present in ≥1 apical view	• Predicted response (sensitivity 95%, specificity 91%)	*n* = 67, prospectively enrolled single cohort. The reported sensitivity of 95% and specificity of 91% were obtained in that same cohort; no validation set was studied [11].	E	[11]
**STE-based LBBB classification**	• Type LBBB-3/4	• Marked LBBB-induced remodelling • Predicts favourable response and super-response	*n* = 250, observational, with supporting experimental data; classification derived within the cohort [65].	E	[65]
**SSI**	• ≥3.1% • ≥2.6% if QRS 120–149 ms	• Freedom from HF hospitalization or death within 2 years	*n* = 442, post hoc analysis of the Adaptive CRT trial cohort. The 3.1% threshold is the cohort median, defined post hoc; the authors call for external validation [15].	O	[15]
**LV mechanical dispersion**	• SD of time to peak negative longitudinal strain across LV segments	• Greater baseline dispersion: higher likelihood of response • Particularly in non-LBBB	*n* = 46, prospective, threshold derived within the cohort [67]; *n* = 278, single-centre prospective, thresholds pre-specified from earlier work [68]. No agreed threshold across studies.	E	[67,68]
**Myocardial work indices**					
**Septal-to-lateral wall work difference**	• ≥860 mmHg%	• Echocardiographic response identified with 75% accuracy • Lower risk of death • Better than QRS duration and morphology, septal flash and apical rocking in that cohort	*n* = 200, prospective multicentre (5 centres), with CMR in 125; the only work-derived variable studied in a prospective multicentre design. Threshold derived within the cohort [17].	E, O	[17]
**GCW**	• >1057 mmHg% combined with GWW > 384 mmHg% • <888 mmHg%	• Combination: 100% specificity and PPV, low sensitivity • <888 mmHg%: cardiac mortality	*n* = 97, single centre, reported in two publications [13,71]; *n* = 166, expanded cohort from the same centre [70]. These are overlapping cohorts, not independent confirmations. All thresholds were derived within the cohort.	E, O	[13,70,71]
**GWW**	• <200 mmHg% (Riolet) • >200 mmHg% (Layec, non-LBBB)	• Low GWW: lower response rate and higher mortality • >200 mmHg% independently predicted response in unfavourable electrical characteristics	*n* = 249, prospective [18]; *n* = 121, retrospective analysis of a prospective registry [20]. Both thresholds were derived within their cohorts; in [20] the ROC-optimal value of 183 mmHg% was rounded to 200.	E, O	[18,20]
**GWE**	• <75% before CRT	• Independently associated with better long-term outcome	*n* = 153, single-centre prospective registry. The 75% value is the cohort median rather than a ROC-derived threshold [16].	O	[16]
**Acute redistribution of regional work**	• Increase in septal and decrease in lateral wall work after CRT	• Determinant of long-term reverse volumetric remodelling	*n* = 130, prospective multicentre; no threshold reported [72].	E	[72]
**Right ventricle**					
**Baseline RV function**	• TAPSE	• Useful predictor of response in meta-analysis • Proposed for future selection algorithms	Study-level meta-analysis of 17 observational studies; the TAPSE analysis pooled 8 studies and 557 patients. The authors explicitly declined to derive a threshold [73].	E	[73]

AVC, aortic valve closure; CMR, cardiac magnetic resonance; CRT, cardiac resynchronization therapy; GCW, global constructive work; GWE, global work efficiency; GWW, global wasted work; HF, heart failure; LBBB, left bundle branch block; LV, left ventricular; LVEF, left ventricular ejection fraction; LVESV, left ventricular end-systolic volume; PPV, positive predictive value; ROC, receiver operating characteristic; RV, right ventricular; SD, standard deviation; SSI, systolic stretch index; STE, speckle-tracking echocardiography; TAPSE, tricuspid annular plane systolic excursion. The two reported GWW thresholds agree in direction but were each derived post hoc within their own cohort; the constructive work thresholds come from overlapping cohorts at a single centre.

## 7. Multimodality Imaging and Myocardial SCAR

Cardiac magnetic resonance (CMR) and nuclear imaging can also assess mechanical dyssynchrony [74,75], but their main contribution to selection is the assessment of scar location and viability, which echocardiography cannot provide.

Larsen et al. showed that septal scar is more strongly associated with non-response than posterolateral scar. Scar in both locations was more common in non-responders, but only septal scar remained an independent marker of non-response in multivariable analysis, suggesting that septal viability is particularly important [22,76]. Duchenne et al., in 170 patients, showed that combining echocardiographic mechanical dyssynchrony (lateral-to-septal work difference, SF, ApRock, SSI) with CMR assessment of septal scar improved the prediction of response and long-term survival. In that study, the highest predictive value was seen when markers of mechanical dyssynchrony were present and septal scar was absent; septal scar itself was an important unfavourable factor. Combined assessment of LV mechanics and septal viability may therefore be more useful than classical parameters alone, such as LVEF, QRS duration or QRS morphology [21].

## 8. Comparative Appraisal of Non-Invasive Markers of Dyssynchrony

The parameters described above are often presented as competing alternatives, but each one assesses a different step in the same chain: the electrical delay, the mechanical dyssynchrony it causes, and the viability of the myocardium that must contract once activation is corrected. Markers of different steps therefore complement rather than replace each other. This section compares the available non-invasive methods by reproducibility, vendor dependence, feasibility in routine practice, added value beyond the standard selection criteria, and level of prospective validation (Table 5).

### 8.1. Quantification of Electrical Dyssynchrony

The 12-lead ECG is the only method whose measures have been validated as selection criteria in randomized trials. QRS duration combined with strict LBBB morphology is therefore the reference against which any other marker must show added value. Automated QRS duration is highly reproducible, but morphology is only moderately so, because the competing LBBB definitions agree poorly [39]. This is a weakness of the reference standard itself, and it limits the added value that any new marker can show.

Vectorcardiographic QRS area is the advanced ECG marker with the largest evidence base. It is calculated automatically from the standard 12-lead ECG by a fixed mathematical transformation, so it is vendor-independent and highly reproducible. It has been studied in independent multicentre cohorts and keeps its predictive value when QRS morphology is unclear, which is exactly where the standard criteria perform worst [59,77]. However, the reported thresholds differ between cohorts, and no randomized trial has compared selection by QRS area with selection by the standard criteria.

Markers of the timing of LV activation (intrinsicoid deflection, QRmax and the Vereckei indices) need only a standard ECG and can be measured at the bedside. However, they are measured by hand in single leads, and their reproducibility depends on correct identification of QRS onset and of the R-wave peak, which is difficult in low-voltage or fragmented complexes. Each comes from a single, fairly small cohort with cut-offs derived in that cohort (Table 3), and none has been externally validated.

Electrocardiographic imaging and related mapping methods measure electrical dyssynchrony directly rather than inferring it from the surface ECG. They need dedicated equipment, body-surface electrode arrays and, for electrocardiographic imaging, a chest computed tomography scan. Their main limitation is feasibility, not physiological validity.

### 8.2. Quantification of Mechanical Dyssynchrony

Visually assessed septal flash and apical rocking are the easiest mechanical markers to use: they need no post-processing, can be seen on standard apical or parasternal views, and do not depend on the echo vendor. Their reproducibility is moderate and depends on the operator, which is the main argument for semi-automated speckle-tracking detection [64]. They have been studied in a multicentre cohort of 1060 patients and are associated with both reverse remodelling and survival [12], but they have never been tested as a selection criterion in a randomized trial.

Strain-based markers are more quantitative but harder to obtain. The classical LBBB strain pattern, the systolic stretch index and mechanical dispersion are less subjective than visual assessment, but all need good image quality, an adequate frame rate and correct timing of valve events; the classical pattern also needs analysis of three apical views. The performance reported for the classical pattern comes from a single-centre cohort of 67 patients in which it was derived [11], and should be read with the same caution as the myocardial work thresholds below.

Myocardial work indices add an estimate of load to strain, but they are the most vendor-dependent parameters considered here: the non-invasive pressure curve, its scaling and the loop segmentation are proprietary and differ between platforms, so absolute values and thresholds cannot be transferred between systems. The two reported thresholds for global wasted work agree in direction but were each derived in their own population [18,20], and most constructive work thresholds come from overlapping cohorts at one centre [13,70,71]. The septal-to-lateral work difference is the most physiologically specific of these indices, because it measures the asymmetry that CRT aims to abolish, and it is the only work-derived variable studied in a prospective multicentre design with a CMR reference [17].

### 8.3. Assessment of the Myocardial Substrate

Late gadolinium enhancement on CMR is the most reproducible of these markers, and the only one that shows directly the tissue responsible for non-response rather than inferring it from the contraction pattern. Its limitations are practical: availability, cost and device-related artefacts in upgrade candidates. No randomized trial has tested scar-guided selection, and the evidence that septal scar predicts non-response comes from observational cohorts [21,22,76].

### 8.4. Association with Response Versus Validation as a Selection Criterion

The key clinical distinction is not between stronger and weaker markers, but between a marker that is associated with response and a marker that has been shown to improve patient selection. Almost all parameters reviewed here belong to the first group. To make this clear, each marker in Table 5 is given one of three levels of validation. Level A: validated as a selection criterion in randomized trials and included in current guidelines (QRS duration, QRS morphology, LVEF and symptoms). Level B: studied prospectively in independent cohorts and shown to predict reverse remodelling or outcome, but never tested as a selection criterion in a randomized trial (vectorcardiographic QRS area, visually assessed septal flash and apical rocking, and septal scar on CMR). Level C: associated with response in single-centre or derivation-only cohorts, with post hoc thresholds and no external validation (all other parameters in Table 5, including all myocardial work indices).

Therefore, no level B or C marker should currently be used to withhold CRT from a patient with a class I indication, or to justify implantation when the guidelines do not support it. Their role is limited to the grey zone where the standard criteria help least (an intermediate QRS duration, a non-specific intraventricular conduction delay or an atypical right bundle branch block) and to an individual discussion of the expected benefit. Going further will require randomized trials that allocate patients according to the marker itself; no such trial exists for any level B or C parameter.

**Table 5:** **Comparative appraisal of non-invasive methods for quantifying electrical and mechanical dyssynchrony and the myocardial substrate.** Level of validation: A, validated as a selection criterion in randomized trials and incorporated into current guidelines; B, evaluated prospectively in independent cohorts as a predictor of response or outcome, but never tested as a randomized selection criterion; C, associated with response in single-centre or derivation-only cohorts, with thresholds defined post hoc and without external validation. Endpoint: E, echocardiographic (reverse remodelling); O, outcome (death and/or heart failure hospitalization); S, selection criterion.

Method	Physiological Target	Principal Metric	Reported Cut-Off	Endpoint	Reproducibility and Vendor Dependence	Level	Major Limitation
12-lead ECG	Electrical dyssynchrony	QRS duration	≥150 ms; 130–149 ms intermediate	S, O	High; automated measurement, vendor-independent	A	Duration is a surrogate for the extent of unopposed activation and ignores its direction
12-lead ECG	Electrical dyssynchrony	QRS morphology (strict LBBB)	Strauss criteria	S, O	Moderate; definition-dependent, only 13.8% concordance between definitions	A	Competing definitions; up to one third of patients labelled LBBB have no true conduction delay
12-lead ECG	Electrical dyssynchrony indexed to cardiac size	QRSd/LVEDV	>0.52 ms/mL	O	High for QRSd; dependent on volumetric measurement	C	Single derivation cohort; requires an accurate LVEDV
12-lead ECG	Timing of LV activation	Intrinsicoid deflection; QRmax; Vereckei indices	ID ≥ 110 ms (I), ≥130 ms (aVL), >60 ms (V6); QRmax > 120 ms; indices > 25%	E	Moderate; manual, lead-specific measurement	C	Derivation-only cohorts; depends on identification of QRS onset and R-wave peak
Vectorcardiography (from 12-lead ECG)	Global electrical dyssynchrony	QRS area	Higher values favourable; thresholds cohort-specific	E, O	High; fixed linear transformation, automated, vendor-independent	B	No randomized trial of selection by QRS area; thresholds differ between cohorts
ECGI, UHF-ECG, electrical dyssynchrony mapping	Activation sequence	Ventricular electrical uncoupling and related indices	Not established	E	Not established outside specialist centres	C	Dedicated hardware, electrode arrays and (for ECGI) thoracic CT; feasibility is the limiting factor
Echocardiography, visual assessment	Mechanical consequence of electrical delay	Septal flash; apical rocking	Present or absent	E, O	Moderate, operator-dependent; vendor-independent	B	Binary and subjective; semi-automated detection improves agreement but is not standard
Speckle-tracking echocardiography	Mechanical consequence of electrical delay	Classical LBBB strain pattern	Three features in ≥1 apical view	E	Moderate; strain values are not interchangeable between vendors	C	Sensitivity 95%/specificity 91% from a single-centre derivation cohort of 67 patients
Speckle-tracking echocardiography	Wasted deformation before and during ejection	Systolic stretch index	≥3.1% (≥2.6% if QRS 120–149 ms)	O	Moderate; requires valve-event timing	C	Derivation-only; depends on frame rate and on timing of aortic valve events
Speckle-tracking echocardiography	Temporal heterogeneity of contraction	Mechanical dispersion	Higher values favourable; no agreed threshold	E	Moderate; vendor- and segment-count dependent	C	Non-specific: elevated in fibrosis and ischaemia without electrical delay
Myocardial work (pressure–strain loops)	Regional work asymmetry	Septal-to-lateral work difference	≥860 mmHg%	E, O	Moderate; strongly vendor-dependent (proprietary pressure curve)	C	Post hoc threshold; values not transferable between platforms
Myocardial work (pressure–strain loops)	Global efficiency of contraction	GCW; GWW; GWE	GCW > 1057 or <888 mmHg%; GWW > 200 mmHg%; GWE < 75%	E, O	Moderate; strongly vendor-dependent	C	Thresholds derived post hoc; the largest series come from overlapping cohorts at one centre
Echocardiography	Right ventricular function	TAPSE	Lower values unfavourable	E	High; simple and vendor-independent	C	Meta-analytic signal only; not evaluated prospectively as a selection variable
CMR with late gadolinium enhancement	Myocardial substrate and viability	Septal scar; total scar burden	Transmural septal scar unfavourable	E, O	High; standardized acquisition, vendor-independent	B	Availability and cost; device artefact in upgrade candidates; scar-guided selection never randomized
Nuclear imaging (FDG-PET, SPECT)	Viability and metabolic activity	Regional viability	Not established for selection	E	High for viability; limited availability	C	Radiation exposure; no incremental value demonstrated over CMR

CMR, cardiac magnetic resonance; CT, computed tomography; ECG, electrocardiogram; ECGI, electrocardiographic imaging; FDG-PET, fluorodeoxyglucose positron emission tomography; GCW, global constructive work; GWE, global work efficiency; GWW, global wasted work; ID, intrinsicoid deflection; LBBB, left bundle branch block; LV, left ventricular; LVEDV, left ventricular end-diastolic volume; QRSd, QRS duration; SPECT, single-photon emission computed tomography; TAPSE, tricuspid annular plane systolic excursion; UHF-ECG, ultra-high-frequency electrocardiography; VCG, vectorcardiography. The two reported GWW thresholds agree in direction but were each derived post hoc within their own cohort; the constructive work thresholds come from overlapping cohorts at a single centre.

## 9. Procedure-Related Factors

The predictors discussed so far are baseline characteristics that identify patients likely to benefit and therefore guide the decision to implant. They should be separated from the procedural and follow-up factors discussed below (LV lead position, percentage of biventricular pacing, atrioventricular (AV) and interventricular (VV) programming, and arrhythmia management), which do not guide selection but determine whether the potential benefit is achieved. Much apparent non-response is due to these modifiable factors rather than to an unfavourable substrate, so a structured review after implantation should come before any conclusion that a patient has not responded.

### 9.1. Left Ventricular Lead Position

Placing the LV lead in the area of latest electrical activation (the Q–LV site) is important for optimal resynchronization [78]. The variable anatomy of the coronary sinus and cardiac veins limits lead positioning. Quadripolar leads offer more pacing configurations, which increases the chance of pacing outside the scar with an acceptable threshold and without phrenic nerve stimulation. However, the randomized DANISH-CRT trial showed that targeting the site of latest electrical activation did not reduce death or first unplanned HF hospitalization compared with conventional non-apical posterolateral placement [79]. Lead position and substrate may still matter, but routine electrical targeting of the latest activated site does not improve outcomes.

### 9.2. Conduction System Pacing

#### 9.2.1. Rationale and Current Evidence

Conduction system pacing (CSP), especially left bundle branch area pacing (LBBAP), may give more physiological activation than conventional biventricular pacing (BiVP), because it uses the native conduction system below the block instead of creating a second, artificial wavefront [80,81]. Reported effects on ECG parameters, LV remodelling and composite endpoints are favourable, and a recent comparison in patients with HFrEF again found LBBAP at least as good as BiVP for surrogate and intermediate endpoints [82]. However, the evidence comes mainly from observational registries, propensity-matched comparisons and a few randomized trials powered for surrogate endpoints (QRS narrowing, LVEF, LV volumes); adequately powered trials with hard clinical endpoints are ongoing. In the randomized LEFT-BUNDLE-CRT trial, LBBAP did not meet non-inferiority to BiVP in the intention-to-treat analysis, although both strategies had high response rates and similar clinical outcomes [83]. A study-level meta-analysis of the available comparisons found similar results for surrogate endpoints and reverse remodelling [84]. LBBAP should therefore not be presented as an established equivalent of biventricular CRT in all populations.

#### 9.2.2. Three Clinical Scenarios

The role of CSP, and the evidence behind it, differ between three clinical situations that are often discussed together but should be kept apart.

The first is CSP as a bail-out after failed coronary sinus lead implantation or an unusable lead position. Here the alternative is no resynchronization at all, the expected benefit is large, and the evidence, although observational, is consistent. This is the best-established use, and it is supported by current guidelines [2,78].

The second is CSP in non-responders to conventional BiV-CRT and in patients upgraded from chronic right ventricular pacing. In both, the electrical problem is at least partly iatrogenic or incompletely corrected, so direct capture of the conduction system makes mechanistic sense. There are no comparative randomized data, and the usual risks of upgrade procedures (venous obstruction, lead extraction, higher infection risk) apply.

The third is CSP as first-line resynchronization in de novo candidates with a class I indication. Here the evidence is weakest compared with the confidence with which it is often presented. LEFT-BUNDLE-CRT did not show non-inferiority, and no trial has yet shown lower mortality or fewer heart failure hospitalizations with LBBAP. Until such data are available, conventional BiV-CRT remains the reference strategy in patients with typical LBBB and a QRS duration of at least 150 ms.

#### 9.2.3. Do the Predictors of Response to Biventricular CRT Apply to Conduction System Pacing?

Whether the predictors reviewed above also apply to CSP is an open question that matters for patient selection, and current studies do not answer it. There are reasons to expect both similarities and differences.

Similar predictive value is expected for markers that show a correctable electrical substrate, whatever the method of correction. Typical LBBB, a large vectorcardiographic QRS area, septal flash, apical rocking and a large septal-to-lateral work difference all show that contraction is disorganized because activation is delayed. This holds whether the delay is corrected by biventricular pacing or by conduction system capture. Septal viability should also remain necessary, since a scarred septum cannot contribute to ejection; however, it is activated.

Differences are expected where the mechanism of correction differs. LBBAP works only if the block lies proximal to the pacing site: a proximal block can be bypassed by left bundle branch area capture, but diffuse distal or Purkinje disease may not be. No routine non-invasive marker can locate the level of block, and this, rather than the degree of dyssynchrony, may turn out to be the main determinant of response to CSP. Extensive septal fibrosis is a second difference, because it affects CSP in two ways: it makes lead fixation and conduction system capture harder, and so reduces procedural success, and it also limits the benefit once capture is achieved. In BiV-CRT, septal scar affects the result but not the feasibility of the implant.

The question for the coming years is therefore not only which patients should receive resynchronization, but which type of resynchronization suits a given electrical and structural substrate. Non-invasive markers that separate a proximal, correctable block from diffuse distal disease, and that measure septal fibrosis before the procedure, would help answer it. No such marker has yet been validated, and the choice between BiVP and CSP remains an individual and largely procedural decision.

#### 9.2.4. Procedural Definition of Successful Left Bundle Branch Area Capture

Any predictor of response to CSP assumes that successful conduction system capture can be defined, because a septal lead without left bundle branch capture delivers myocardial, not conduction system, pacing. The EHRA clinical consensus statement combines paced QRS morphology, the timing of LV activation and a visible transition between capture types [85]. A right bundle branch delay pattern in lead V1 is expected during left bundle branch area capture. The stimulus-to-LV activation time (from the pacing stimulus to the R-wave peak in V5–V6) should be short and should either stay constant at high and low output or shorten suddenly at the transition from non-selective to selective capture. The interval between the R-wave peaks in V6 and V1 has been proposed as a quantitative criterion to separate left bundle branch capture from septal myocardial capture [86]. Paced QRS duration alone is not enough, because a narrow paced QRS can also result from well-placed septal myocardial pacing.

The link between these procedural criteria and later mechanical resynchronization has been studied much less than the electrical criteria themselves. The mechanical counterparts (resolution of septal flash and apical rocking, loss of the septal-to-lateral work difference, and a shift in regional work towards the septum) can be measured soon after implantation and are the logical link between a technically successful implant and reverse remodelling at follow-up. Studies linking the electrical capture criteria to these acute mechanical endpoints, and both to remodelling at 6–12 months, are needed to show whether the current electrical definitions identify the physiologically relevant result.

#### 9.2.5. Other Pacing Configurations

Two other configurations are relevant. His bundle pacing corrects conduction at the most proximal level and is the most physiological option, but it is limited by lead instability, higher and often rising capture thresholds and a lower implant success rate; it is now used mainly when left bundle branch area capture cannot be achieved [87]. Multipoint pacing uses two LV pacing vectors from a quadripolar lead. Large randomized trials have not clearly shown that it is better than standard biventricular pacing in unselected patients, and it increases battery use, has less favourable pacing thresholds and lacks a standard programming algorithm [88]. With any configuration, pacing within scar should be avoided, because it limits the benefit; however, activation is delivered.

### 9.3. Programming

A key goal is a high percentage of biventricular (BiV) pacing, ideally close to 100%. About 40% of CRT recipients do not reach this goal because of atrial tachyarrhythmias, AF or frequent premature ventricular complexes. In HF patients with paroxysmal or persistent AF, catheter ablation is better than drug therapy at maintaining sinus rhythm and has been associated with better outcomes; however, this evidence does not come specifically from CRT recipients, so its use to restore a high percentage of biventricular pacing is indirect [89]. In permanent AF with a fast ventricular rate, atrioventricular junction ablation may increase BiV pacing.

If there is no clinical improvement despite optimal drug therapy and a high percentage of BiV pacing, the AV and VV delays should be optimized [90,91]. Modern devices have automatic algorithms (e.g., SyncAV, AdaptivCRT, Autoadapt) to improve atrioventricular and interventricular synchrony [92]. Optimization using echocardiographic parameters (mitral inflow, LV outflow tract velocity–time integral [LVOT VTI], stroke volume, septal-to-lateral delay, longitudinal strain) has been widely studied, with disappointing results. Routine AV delay optimization is not recommended, but it may be considered in non-responders. A small randomized trial in 30 non-responders showed that AV and VV programming guided by STE strain patterns improved LVEF and NYHA class compared with conventional optimization [93], but there are no large trials of this method.

### 9.4. Arrhythmia Management and Post-Implantation Follow-Up

Loss of effective resynchronization during follow-up is a common and often reversible cause of non-response. Atrial tachyarrhythmias, frequent premature ventricular complexes and a too-long sensed AV delay all reduce the proportion of truly resynchronized beats, and device counters may overestimate this proportion because fusion and pseudo-fusion beats are counted as paced. In permanent AF with poor rate control, atrioventricular junction ablation increases effective biventricular pacing [94]; in frequent premature ventricular complexes, catheter ablation or antiarrhythmic drugs may restore it [95]. Every patient should therefore be reviewed in a device and heart failure clinic before being called a non-responder, with checking of true biventricular capture, lead position and pacing vector, reprogramming where needed, and further optimization of guideline-directed medical therapy.

## 10. CRT Upgrade

The evidence discussed so far concerns de novo implantation. However, many procedures are upgrades from a pacemaker or ICD in patients who develop HF during chronic right ventricular pacing. Right ventricular apical or septal pacing produces an LBBB-like LV activation pattern, and a pacing burden above about 20% is associated with progressive LV dysfunction and pacing-induced cardiomyopathy (PICM) [96]. Until recently, evidence for upgrade came from small observational studies and registries, and the 2021 ESC guidelines therefore downgraded the recommendation from class I to class IIa [2].

The BUDAPEST-CRT Upgrade trial has since provided randomized evidence [97]. It randomized 360 patients with a previously implanted device, LVEF ≤ 35%, NYHA class II–IV, a paced QRS ≥ 150 ms without intrinsic LBBB and a right ventricular pacing burden ≥ 20% to a CRT-D upgrade or ICD therapy. Over a median of 12.4 months, the primary composite outcome occurred in 32.4% of the upgrade arm versus 78.9% of the ICD arm (odds ratio 0.11; 95% CI 0.06–0.19), with a consistent effect in all prespecified subgroups, much more reverse remodelling, and numerically more procedure- or device-related complications after upgrade.

Two points matter for predicting response. First, the predictors differ from those in de novo patients. The dyssynchrony is iatrogenic and therefore correctable, which may partly explain the very large effect in BUDAPEST-CRT. Intrinsic QRS morphology, central to de novo selection, is of little value in a chronically paced patient; paced QRS duration and right ventricular pacing burden are the relevant parameters instead. Second, upgrade carries extra risks, including venous obstruction, lead extraction and a higher rate of pocket infection. These should be weighed against the expected benefit, and the upgrade should not simply be delayed until elective generator replacement. Conduction system pacing is increasingly used instead of a coronary sinus lead in this setting, although randomized comparisons are not yet available.

## 11. Artificial Intelligence

Given the many possible predictors and their complex interactions, a unified risk model may require machine learning and artificial intelligence (AI), which can integrate large and varied clinical, electrophysiological and imaging data. However, current models come mostly from retrospective registries and post hoc analyses of trial data, are prone to overfitting, and have rarely been externally validated or tested prospectively; none has been shown to improve outcomes, and none is recommended by guidelines. Machine learning should therefore be seen as a research tool, not a clinical selection tool. In the future, computational “digital twin” models may allow the effect of therapy to be simulated in an individual patient, supporting more personalized treatment [98,99,100].

## 12. Conclusions

The proportion of patients who do not respond to CRT remains high. The evidence reviewed here shows that the greatest benefit is obtained when mechanical dyssynchrony is caused by delayed electrical activation and the septum is viable. Because several mechanisms contribute to response, no single parameter identifies responders, and a multimodal assessment combining clinical, electrocardiographic and imaging data is more likely to succeed. The predictors discussed here cannot be ranked against one another: they were studied in different populations, against different endpoints and at different time points, and head-to-head comparisons are largely lacking.

Based on the evidence reviewed above, we propose a practical framework for everyday use (Figure 4). It is organized by the clinical question rather than by the technique, and it separates four domains that are often mixed up. In each domain, parameters that are part of current guideline recommendations are separated from those that still need prospective evaluation. The framework is a way to structure the assessment, not a validated decision rule, and investigational markers should not be read as indications for treatment.

Eligibility. Confirm symptomatic heart failure despite at least three months of optimized guideline-directed medical therapy and an LVEF of 35% or less, and record the underlying rhythm and the expected ventricular pacing burden. These are guideline criteria, not predictors of response.

Domain 1—Electrical dyssynchrony. Assess QRS morphology using strict LBBB criteria and QRS duration. In patients with a class I indication (typical LBBB with a QRS duration of at least 150 ms), this is enough, and no further testing is needed before implantation. In patients with an intermediate or uncertain indication (a QRS duration of 130–149 ms, a non-specific intraventricular conduction delay or an atypical right bundle branch block), markers of delayed LV activation (intrinsicoid deflection, QRmax, vectorcardiographic QRS area) may add information. Of these, only QRS area has been studied prospectively in independent cohorts, and none has been tested as a selection criterion in a randomized trial.

Domain 2—Mechanical dyssynchrony. In the same intermediate group, check whether the electrical delay has caused a correctable mechanical dyssynchrony. Septal flash and apical rocking are the easiest markers to use and have prospective prognostic data. The classical LBBB strain pattern, the systolic stretch index, mechanical dispersion and the septal-to-lateral work difference measure the same phenomenon but remain investigational: their thresholds come from single cohorts, and work-derived values cannot be transferred between vendors.

Domain 3—Myocardial substrate and viability. Assess scar burden and septal viability, preferably by CMR and especially in ischaemic etiology. Septal scar is consistently associated with non-response in observational cohorts and also helps to guide lead placement. Preserved septal viability combined with mechanical dyssynchrony identifies the substrate in which CRT is most likely to work; scar-guided selection has not been tested in a randomized trial.

Domain 4—Choice of resynchronization modality. Choose between conventional biventricular pacing and conduction system pacing. Biventricular pacing remains the reference strategy for patients with a class I indication. Conduction system pacing is best established as a bail-out after failed coronary sinus lead implantation, and is a reasonable option in non-responders, in upgrades from chronic right ventricular pacing and when a high ventricular pacing burden is expected; its role as first-line therapy in de novo candidates is not established. Whether the markers in domains 1 to 3 predict response to conduction system pacing as they do for biventricular pacing is unknown; this is currently the largest gap between the evidence and clinical practice.

Procedure and follow-up. Avoid pacing in areas of significant scar where possible, aim for a stable non-apical LV lead position with good electrical parameters, and consider quadripolar leads. During follow-up, confirm true biventricular capture close to 100%, treat arrhythmias that reduce it, consider AV and VV delay optimization in non-responders, and continue up-titration of medical therapy before concluding that the patient has not responded.

## Figures and Tables

**Figure 1:**
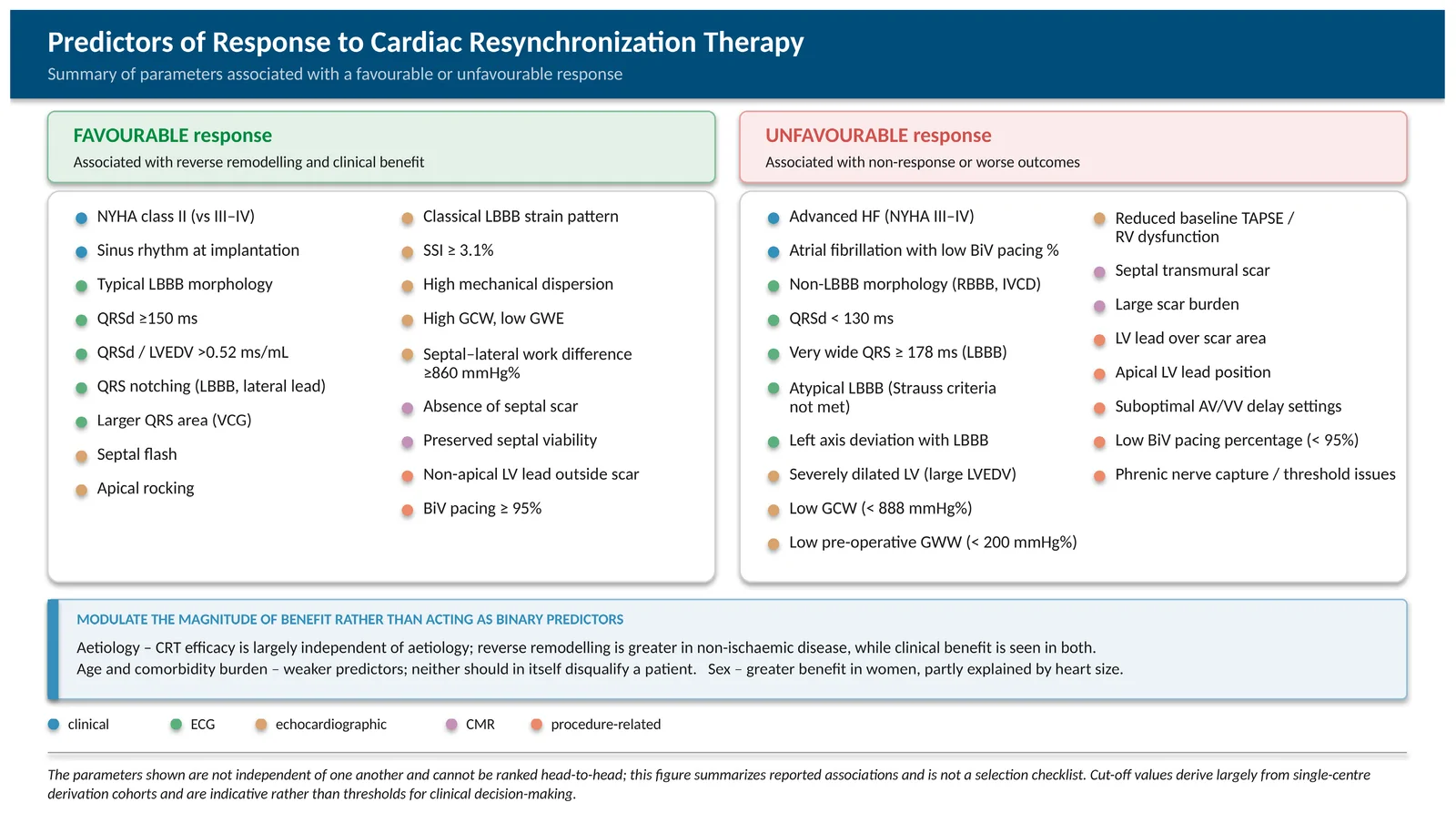
Parameters associated with a favourable or unfavourable response to CRT. Marker colour shows the category: clinical (blue), ECG (green), echocardiographic (yellow), CMR (purple), procedure-related (orange). Etiology, age, comorbidity burden and sex are shown in the lower band, because they change the size of the benefit rather than acting as yes/no predictors: CRT efficacy is largely independent of etiology, with more reverse remodelling in non-ischaemic disease but clinical benefit in both groups, and neither older age nor more comorbidities should by themselves exclude a patient. The parameters are not independent of each other and cannot be ranked head-to-head; the figure summarizes reported associations and is not a selection checklist. Abbreviations: AV/VV, atrioventricular/interventricular; BiV, biventricular; CMR, cardiac magnetic resonance; GCW, global constructive work; GWE, global work efficiency; GWW, global wasted work; HF, heart failure; IVCD, non-specific intraventricular conduction delay; LBBB, left bundle branch block; LV, left ventricle; LVEDV, left ventricular end-diastolic volume; NYHA, New York Heart Association; QRSd, QRS duration; RBBB, right bundle branch block; RV, right ventricle; SSI, systolic stretch index; TAPSE, tricuspid annular plane systolic excursion; VCG, vectorcardiography.

**Figure 2:**
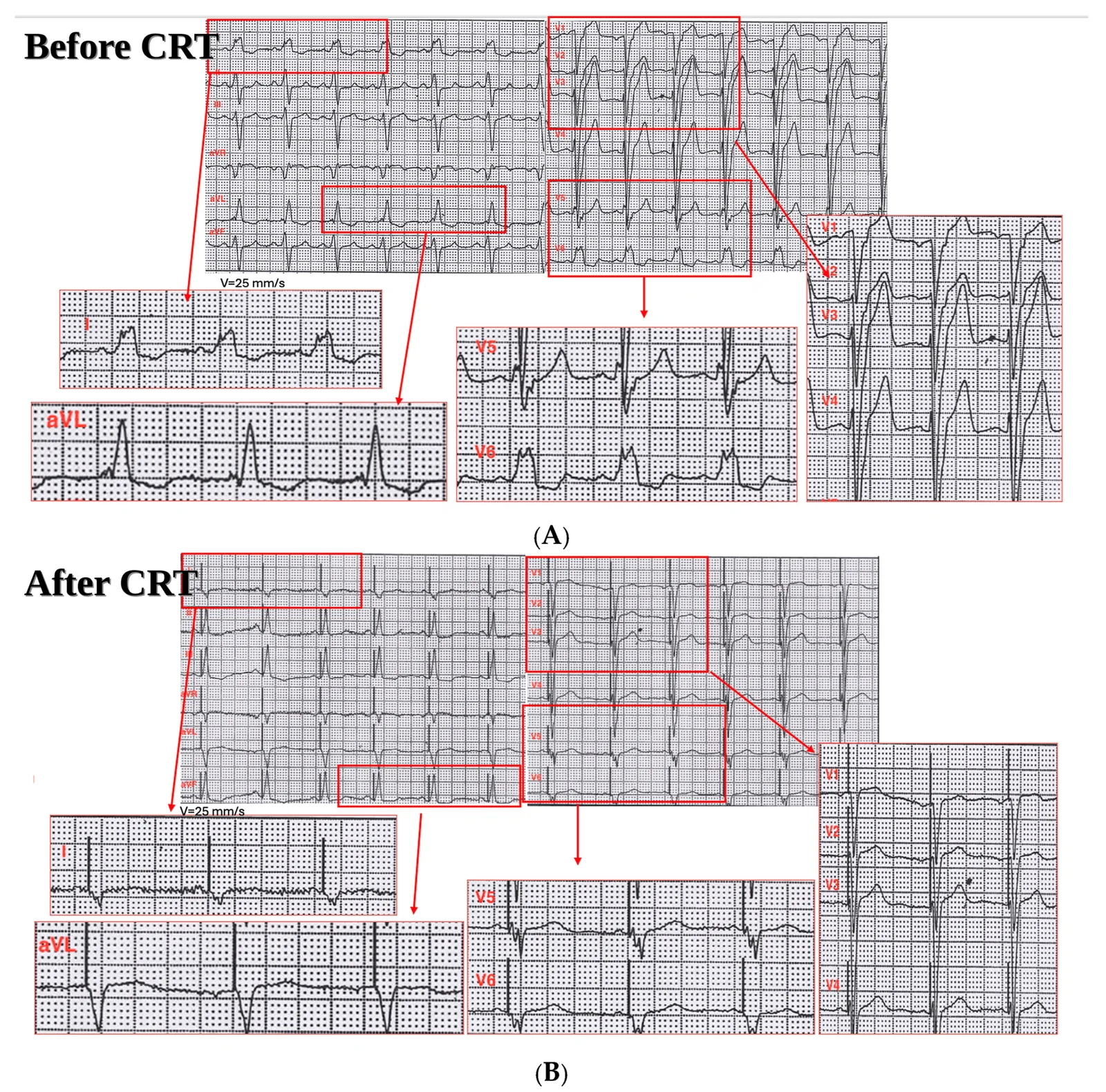
(**A**) ECG before CRT implantation in a male patient fulfilling the strict criteria for left bundle branch block according to Strauss et al. The QRS duration was 170 ms and the PR interval was 160 ms (automatic measurements). Paper speed: 25 mm/s; calibration: 10 mm/mV. (**B**) ECG after CRT implantation showing QRS narrowing from 170 to 140 ms (automatic measurement). Paper speed: 25 mm/s; calibration: 10 mm/mV.

**Figure 3:**
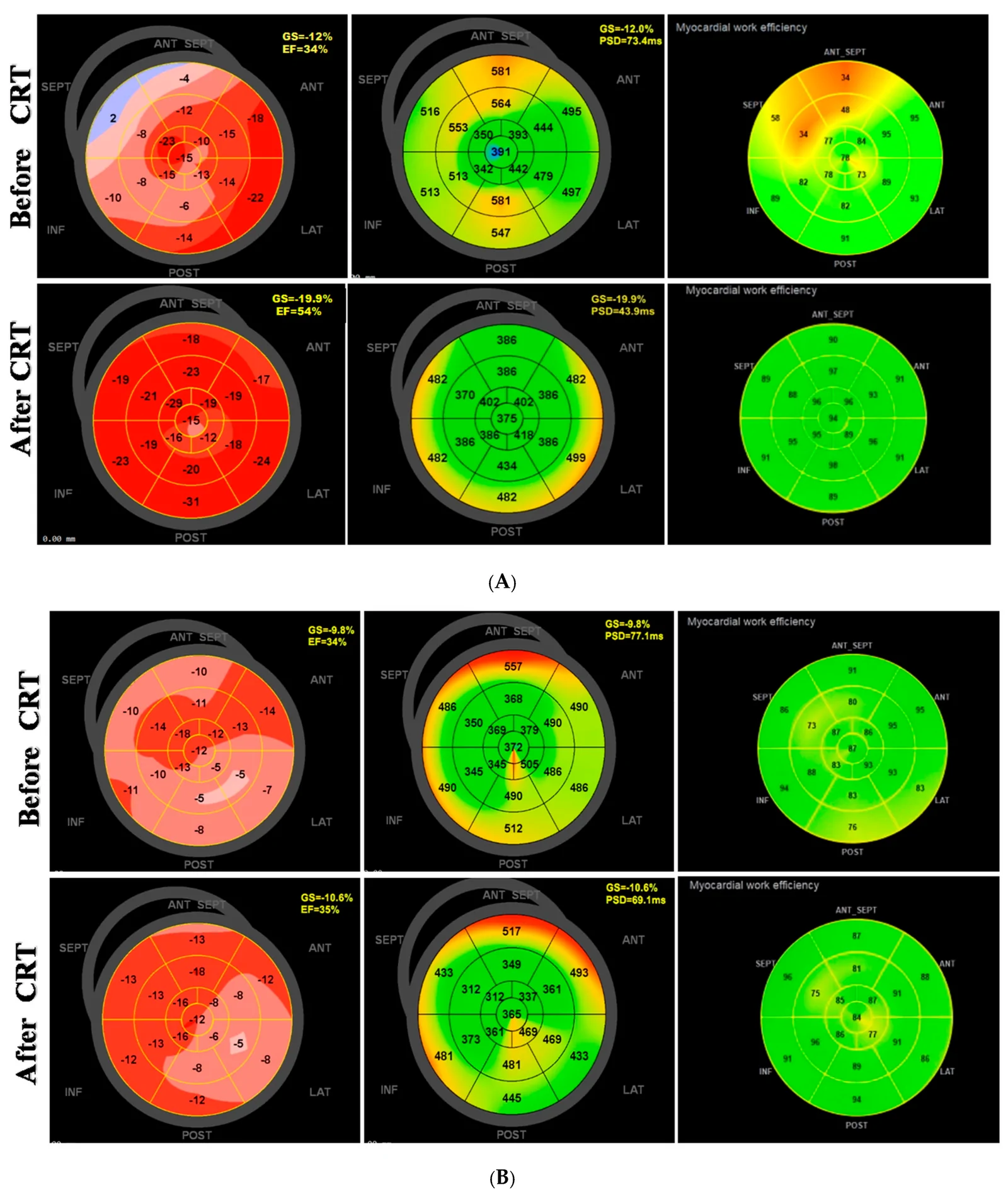
(**A**) Favourable echocardiographic response to CRT. Female, 68 years, non-ischaemic cardiomyopathy, sinus rhythm, typical LBBB, baseline QRS 160 ms. Bull’s-eye maps of segmental longitudinal strain (**left**), time to peak longitudinal strain (**centre**) and myocardial work efficiency (**right**), before (**top**) and 12 months after (**bottom**) CRT implantation. Global longitudinal strain improved from −12.0% to −19.9% and peak strain dispersion decreased from 73.4 ms to 43.9 ms. LVEF increased from 34% to 54% and LVESV decreased from 86 mL to 38 mL (−56%), fulfilling the criterion of echocardiographic response (LVESV reduction ≥ 15%). Abbreviations: CRT, cardiac resynchronization therapy; GS, global longitudinal strain (EchoPAC notation); LBBB, left bundle branch block; LVEF, left ventricular ejection fraction; LVESV, left ventricular end-systolic volume. (**B**) Unfavourable echocardiographic response to CRT. Male, 60 years, non-ischaemic cardiomyopathy, sinus rhythm, typical LBBB, baseline QRS 134 ms. Bull’s-eye maps of segmental longitudinal strain (**left**), time to peak longitudinal strain (**centre**) and myocardial work efficiency (**right**), before (**top**) and 12 months after (**bottom**) CRT implantation. Global longitudinal strain changed only marginally, from −9.8% to −10.6%, peak strain dispersion decreased from 77.1 ms to 69.1 ms, and LVEF was essentially unchanged (34% to 35%). Despite these small changes in the parameters shown, LVESV increased from 118 mL to 135 mL (+14%), and the patient was classified as a non-responder. Of note, this patient had a QRS duration of 134 ms with typical LBBB, corresponding to a class IIa indication, whereas the patient in Figure 3A fulfilled a class I indication (typical LBBB with QRS 160 ms); together, the two cases illustrate that QRS duration and morphology alone do not reliably predict the individual response, and that baseline global longitudinal strain, the degree of left ventricular remodelling and sex may carry additional information. Abbreviations as in Figure 3A. Analyses were performed using EchoPAC (GE HealthCare).

**Figure 4:**
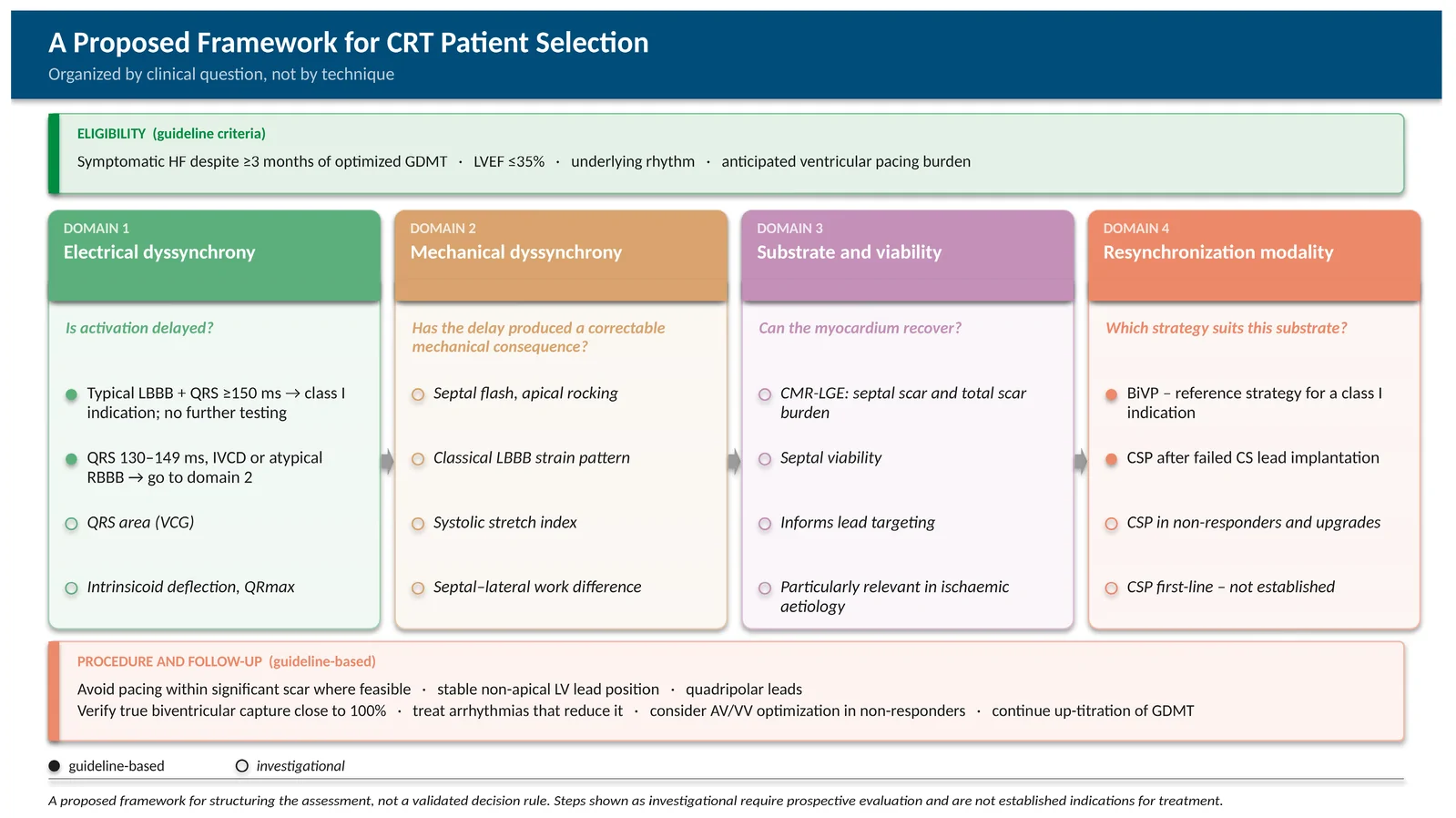
A proposed framework for CRT patient selection, organized into four domains: electrical dyssynchrony, mechanical dyssynchrony, myocardial substrate and viability, and choice of resynchronization modality. Filled markers show steps that are part of current guideline recommendations; open markers in italics show markers that need prospective evaluation and are not established indications for treatment. The framework is a way to structure the assessment, not a validated decision rule. Abbreviations: AV, atrioventricular; BiVP, biventricular pacing; CMR, cardiac magnetic resonance; CRT, cardiac resynchronization therapy; CS, coronary sinus; CSP, conduction system pacing; GDMT, guideline-directed medical therapy; HF, heart failure; IVCD, non-specific intraventricular conduction delay; LBBB, left bundle branch block; LGE, late gadolinium enhancement; LV, left ventricular; LVEF, left ventricular ejection fraction; RBBB, right bundle branch block; VCG, vectorcardiography; VV, interventricular.

**Table 1:** **Indications for cardiac resynchronization therapy according to the 2021 ESC Guidelines on cardiac pacing and cardiac resynchronization therapy [2].** Applies to all rows unless stated otherwise: symptomatic HF (NYHA class II–IV) despite ≥3 months of OMT, LVEF ≤ 35%, sinus rhythm.

Clinical Setting/Criteria	Class/Level
**Sinus rhythm—QRS morphology and duration**	
• QRS ≥ 150 ms • LBBB	I A
• QRS 130–149 ms • LBBB	IIa B
• QRS ≥ 150 ms • Non-LBBB	IIa B
• QRS 130–149 ms • Non-LBBB	IIb B
• QRS < 130 ms • No indication for ventricular pacing • CRT not indicated	III A
**Atrial fibrillation**	
• Permanent AF • NYHA III or ambulatory IV • Intrinsic QRS ≥ 130 ms • Strategy to ensure biventricular capture in place	IIa C
• Uncontrolled ventricular rate • Undergoing AV junction ablation • Any QRS duration	I B
**Indication for ventricular pacing or upgrade (LVEF criterion as stated)**	
• High-degree AV block • LVEF < 40% • CRT rather than RV pacing • Includes patients with AF	I A
• Conventional pacemaker or ICD recipient • Symptomatic HF with high proportion of RV pacing (PICM) • Upgrade to CRT	IIa B
• Coronary sinus lead implantation unsuccessful • His bundle pacing as an alternative	IIa B

AF, atrial fibrillation; AV, atrioventricular; CRT, cardiac resynchronization therapy; HF, heart failure; ICD, implantable cardioverter-defibrillator; LBBB, left bundle branch block; LVEF, left ventricular ejection fraction; NYHA, New York Heart Association; OMT, optimal medical therapy; PICM, pacing-induced cardiomyopathy; RV, right ventricular. Class/Level denotes the class of recommendation followed by the level of evidence. The choice between CRT-P (CRT pacemaker) and CRT-D (CRT defibrillator) follows the separate indications for defibrillator therapy and is not part of the resynchronization indication itself.

**Table 2:** **Definitions of CRT response used in the key studies cited in this review. The table lists studies whose own definition of response is discussed in the text; studies cited only for a reported association are summarized in Table 3 and Table 4.** “Echo response” denotes a reduction in LVESV ≥ 15% unless stated otherwise. Type of endpoint: E, echocardiographic; C, clinical; O, outcome (death and/or HF hospitalization).

Study (Year) [Ref.]	Design	*n*	Endpoint	Timing	Type
Fornwalt (2010) [8]	Post hoc analysis, PROSPECT	426	• 15 published definitions compared	6 mo	E, C
Risum (2012) [11]	Prospective cohort	67	• Echo response	6 mo	E
Stankovic (2016), PREDICT-CRT [12]	Retrospective, multicentre	1060	• Echo response • All-cause mortality	Echo 12 ± 6 mo; death 46 mo	E, O
Galli (2018) [13]	Retrospective cohort	97	• Echo response	6 mo	E
Zweerink (2018) [14]	Retrospective, two centres	250	• Death, LVAD or transplantation	≈4 y	O
Gorcsan (2019), SSI [15]	Adaptive CRT trial cohort	442	• HF hospitalization or death • All-cause death	2 y	O
Van der Bijl (2019) [16]	Observational registry	153	• Echo response • All-cause mortality	Echo 6 mo; death 57 mo	E, O
Aalen (2020) [17]	Prospective, multicentre	200	• Echo response • Survival free of heart transplantation	Echo 6 mo; outcome 35 ± 11 mo	E, O
Riolet (2021) [18]	Prospective cohort	249	• Echo response and/or no HF hospitalization • All-cause mortality	6 mo; death 48 mo	E, C, O
Friedman (2023) [19]	Meta-analysis, patient-level	6264	• HF hospitalization or death • All-cause mortality	24 mo	O
Layec (2023) [20]	Retrospective cohort	121	• Echo response • Death or HF hospitalization	9 mo	E, O
Duchenne (2023) [21]	Prospective, multicentre	170	• Echo response • All-cause mortality	12 mo	E, O
Larsen (2023) [22]	Prospective, multicentre	136	• Echo response • Transplantation or all-cause mortality	Echo 6 mo; outcome 39 ± 13 mo	E, O
Friedman (2024) [23]	Meta-analysis, patient-level	5628	• HF hospitalization or death • All-cause mortality	Trial-dependent	O
Sudesh (2024) [24]	Meta-analysis, 7 RCTs	6252 (echo 2430)	• Death or HF hospitalization • All-cause mortality • LVEF, LVEDV, LVESV change	Echo 12 mo; clinical 20 mo	E, O
Yamamoto (2024) [25]	Retrospective, multicentre	119	• HF hospitalization • All-cause mortality, LVAD • Echo response	Echo 6 mo; outcome 878 d	E, O

CRT, cardiac resynchronization therapy; d, days; echo, echocardiographic; HF, heart failure; LVAD, left ventricular assist device; LVEDV, left ventricular end-diastolic volume; LVEF, left ventricular ejection fraction; LVESV, left ventricular end-systolic volume; mo, months; RCT, randomized controlled trial; SSI, systolic stretch index; y, years. Studies without an E code did not apply a binary echocardiographic responder definition.

## Data Availability

No new data were created or analyzed in this study. Data sharing is not applicable to this article.

## Supplementary Materials

The following supporting information can be downloaded at: https://www.mdpi.com/article/10.3390/biomedicines14102239/s1, Supplementary Materials S1: Literature search strategy.

## Abbreviations

AF	atrial fibrillation
AI	artificial intelligence
ApRock	apical rocking
AV	atrioventricular
BiV	biventricular
BiVP	biventricular pacing
BSA	body surface area
CMR	cardiac magnetic resonance
CRT	cardiac resynchronization therapy
CSP	conduction system pacing
ECG	electrocardiogram
ECGI	electrocardiographic imaging
GCW	global constructive work
GDMT	guideline-directed medical therapy
GWE	global work efficiency
GWI	global work index
GWW	global wasted work
HF	heart failure
HFrEF	heart failure with reduced ejection fraction
ICD	implantable cardioverter-defibrillator
ICM	ischaemic cardiomyopathy
ID	intrinsicoid deflection
IVCD	non-specific intraventricular conduction delay
LA	left atrium
LBBAP	left bundle branch area pacing
LBBB	left bundle branch block
LOR	lead one ratio
LV	left ventricle
LVAD	left ventricular assist device
LVEF	left ventricular ejection fraction
LVEDV	left ventricular end-diastolic volume
LVESV	left ventricular end-systolic volume
LVOT	left ventricular outflow tract
MW	myocardial work
NICM	non-ischaemic cardiomyopathy
NYHA	New York Heart Association
PICM	pacing-induced cardiomyopathy
PPV	positive predictive value
PSL	pressure–strain loop
QRSd	QRS duration
RBBB	right bundle branch block
RV	right ventricle
SCD	sudden cardiac death
SD	standard deviation
SF	septal flash
SSI	systolic stretch index
STE	speckle tracking echocardiography
TAPSE	tricuspid annular plane systolic excursion
VCG	vectorcardiography
VTI	velocity–time integral
VV	interventricular
