# Supplementary material for: Predictors of Favourable Response to Cardiac Resynchronization Therapy in Patients with Heart Failure with Reduced Ejection Fraction: A Narrative Review
Source: Biomedicines. 2026 Oct 3;14(10):2239. doi: 10.3390/biomedicines14102239 (PMC13701934; doi:10.3390/biomedicines14102239)
Supplement: Supplementary file 1 [file biomedicines-14-02239-s001.zip › biomedicines-4593082-supplementary.pdf]

Supplementary Material S1

Predictors of favourable response to cardiac resynchronization therapy in patients with heart failure with reduced ejection fraction: a narrative review

Magdalena Potapowicz-Krysztofiak, Małgorzata Maciorowska, Beata Uziębło-Życzkowska

Literature search strategy

Databases and time period. Relevant articles were identified by searching PubMed and Google Scholar from database inception up to September 2026.

Search strings. Three blocks of terms were combined with AND:

- ("cardiac resynchronization therapy" OR "CRT" OR "biventricular pacing" OR "conduction system pacing" OR "left bundle branch area pacing")
- AND ("responder" OR "non-responder" OR "response" OR "reverse remodelling" OR "reverse remodeling")
- AND ("predictor" OR "prediction" OR "patient selection")

The first two blocks were also combined with each of the following:

- ("dyssynchrony" OR "septal flash" OR "apical rocking" OR "mechanical dispersion" OR "systolic stretch")
- ("myocardial work" OR "pressure-strain loop" OR "constructive work" OR "wasted work")
- ("QRS duration" OR "QRS morphology" OR "left bundle branch block" OR "QRS area" OR "vectorcardiography" OR "intrinsicoid deflection")
- ("scar" OR "viability" OR "late gadolinium enhancement" OR "cardiac magnetic resonance")

Both the British and the American spelling of “remodelling” were used.

Additional sources. Reference lists of the retrieved articles and of recent guidelines were also screened.

Inclusion criteria. Priority was given to original studies, meta-analyses, guidelines and consensus documents published in peer-reviewed journals in which response to CRT, reverse remodelling or clinical outcome was an endpoint. Review articles were cited for background and context.

Exclusion criteria. Case reports and paediatric studies were not included. Conference abstracts, articles not in English and studies with only acute haemodynamic endpoints were generally not included; they were cited only exceptionally, when they added mechanistic or practical information not available elsewhere.

Selection. Articles were selected on the basis of clinical relevance and the authors' expertise, with priority given to randomized trials, patient-level meta-analyses and multicentre studies. As this is a narrative review, no protocol was registered, and no formal study-selection process or risk-of-bias assessment was applied. Overlapping cohorts are identified in the text and tables of the main manuscript.

CRT, cardiac resynchronization therapy.
